# Application of the radial artery after angiography in patients undergoing total arterial coronary revascularization

**DOI:** 10.1186/s13019-024-02893-7

**Published:** 2024-07-03

**Authors:** Zanxin Wang, Haibing Liu, Zhifu Huan, Chao Su, Yao Chen, Minxin Wei

**Affiliations:** Department of Cardiac Surgery, The University of Hong Kong-Shenzhen Hospital, Guangdong, P.R. China

**Keywords:** Coronary artery bypass grafting surgery, Radial artery, Ultrasound test

## Abstract

**Objective:**

There is growing evidence supporting the utilization of the radial artery as a secondary arterial graft in coronary artery bypass grafting (CABG) surgery. However, debates continue over the recovery period of the radial artery following angiography. This study aims to evaluate the clinical outcomes and experiences related to the use of the radial artery post-angiography in total arterial coronary revascularization.

**Methods:**

A retrospective analysis was performed on data from patients who underwent total arterial CABG surgery at the University of Hong Kong Shenzhen Hospital from July 1, 2020, to September 30, 2022. Preoperative assessments included ultrasound evaluations of radial artery blood flow, diameter, intimal integrity, and the Allen test. Additionally, pathological examinations of the distal radial artery and coronary artery CT angiography were conducted, along with postoperative follow-up to assess the safety and efficacy of using the radial artery in patients undergoing total arterial CABG.

**Results:**

A total of 117 patients, compromising 102 males and 15 females with an average age of 60.0 ± 10.0 years, underwent total arterial CABG. The internal mammary artery was used in situ in 108 cases, while in 4 cases, it was grafted to the ascending aorta due to length limitations. Bilateral radial arteries were utilized in 88 patients, and bilateral internal mammary arteries in 4 patients. Anastomoses of the proximal radial arteries to the proximal ascending aorta included 42 cases using distal T-anastomosis and 4 using sequential grafts. The interval between bypass surgery and coronary angiography ranged from 7 to 14 days. Pathological examination revealed intact intima and continuous elastic membranes with no significant inflammatory infiltration or hyperplastic lumen stenosis in the radial arteries. There were no hospital deaths, 3 cases of perioperative cerebral infarction, 1 secondary thoracotomy for hemorrhage control, 21 instances of intra-aortic balloon pump (IABP) assistance, and 2 cases of poor wound healing that improved following debridement. CT angiography performed 2 weeks post-surgery showed no internal mammary artery occlusions, but 4 radial artery occlusions were noted.

**Conclusion:**

Ultrasound may be used within 2 weeks post-angiography to assess the recovery of the radial artery in some patients. Radial arteries with intact intima may be considered in conjunction with the internal mammary artery for total arterial coronary CABG. However, long-term outcomes of these grafts require further validation through larger prospective studies.

## Background

After CABG surgery, numerous studies have confirmed the long-term patency advantage of arterial grafts [1–3]. The radial artery, initially proposed by Carpentier as a graft candidate for CABG surgery, offers advantages such as easy clinical evaluation and harvest, a larger diameter, and quicker wound recovery compared to veins from the lower limbs [4]. However, it was temporarily abandoned due to the risk of spasm [5]. With the introduction of antispasmodic drugs and advancements in surgical techniques, an increasing number of studies have shown that total arterial coronary artery bypass grafting surgery leads to a better long-term survival rate and a lower incidence of cardiovascular events [6, 7]. Thus, the radial artery remains a preferred choice as the second graft in arterial bypass grafting.

Currently, the left radial artery approach for coronary angiography has become a standard procedure, with most studies reporting a four-week recovery period for the distal intima of the artery following puncture [8, 9]. However, at our center, complete recovery of the radial artery’s intima, as assessed by ultrasound, has been observed within 7–14 days after angiography. Consequently, this study aims to analyze the timing, safety, and reliability of utilizing the radial artery in total artery bypass grafting surgery.

## Materials and methods

### General information

A retrospective analysis was conducted on data from patients who underwent total arterial coronary artery bypass grafting surgery at the University of Hong Kong Shenzhen Hospital between July 1, 2020, and September 30, 2022. The inclusion criteria were patients who utilized the radial and internal mammary arteries for the surgery. The exclusion criteria were: (1) patients with venous grafts and (2) those who underwent secondary surgery. Patient demographics, graft vessels, target vessels, and follow-up results were recorded.

### Radial artery evaluation

For patients who underwent coronary angiography via the right radial artery, detailed evaluations of intimal smoothness and thromboembolism at the distal end was performed. There is a specialized ultrasound physician who evaluates all radial artery intima and completes the Allen’s test to ensure consistency in the study.

The ultrasound Doppler was enhanced by introducing additional measurement indices to provide a more accurate evaluation of the radial artery, especially after use of the right radial artery for angiography. (1) Evaluation of radial artery diameter: measurements were taken at the radial styloid process, 2 cm below the elbow fossa, and the midsection of the radial artery to accurately assess vessel thickness and determine suitability for use as a bypass graft. (2) Ultrasonic ALLEN test: Traditional ALLEN tests can be influenced by some factors including the completeness of radial artery compression, skin color, and lighting conditions, potentially leading to inaccuracies. Ultrasound can eliminate these variables. A 30% increase in ulnar artery blood flow was considered indicative of adequate collateral circulation in the palm. (3) Evaluation of intima: In addition to diameter, evaluating the intima of the artery is crucial.

If a thrombus was detected, ultrasound determined the suitable length for bypass and marked it on the body surface.

The radial artery was harvested using the “NO-TOUCH” technique via an open incision to minimize damage [10] and stored in a solution of papaverine and heparin saline. After thoracotomy, the artery was placed in the pericardium for heat preservation.

### Pathology

The scar or thrombus at the distal end of the radial artery was removed, and a smooth and intact part of the intima was taken as a graft vessel. The sample was taken from the normal vascular part to confirm the integrity of the intima at the anastomotic site.

All samples were fixed in 4% paraformaldehyde overnight, then embedded in paraffin, and sliced into 3 μm sections. After routine dewaxing and dehydration, standard hematoxylin and eosin (HE) staining was performed, including 10 min of hematoxylin staining, decolorization with 1% dilute hydrochloric acid for 5 s, treatment with 0.01 mmol/L PBS (pH 7.2–7.4) for 5 min, and flushing with tap water for 2 min after each step. The eosin solution was applied for 30 s to 1 min, followed by dehydration with 70-100% gradient alcohol for 1–2 min, xylene clarification, and sealing with neutral gum. The vascular structure and intimal integrity of the radial artery were assessed under an optical microscope using a double-blind method.

### Surgical strategy

Based on preoperative cardiac function, 99 patients underwent off-pump CABG (OPCAB), 9 patients received bypass surgery on a beating heart assisted by cardiopulmonary bypass, and 15 patients underwent on-pump surgery for mitral valve or ventricular aneurysm or other intracardiac operations. The anastomosis sequence began with the radial artery anastomosed to the ascending aorta using 7 − 0 prolene. After confirming unobstructed blood flow, the distal end of the radial artery was clamped to pre-adapt it to aortic pressure. Then, the anastomosis from the internal mammary artery to the left anterior descending branch, and the anastomosis of the distal radial artery were completed, allowing the radial artery sufficient time to regain natural tension. The typical size of an anastomosis was approximately 6 mm.

### Statistical analysis

Data were analyzed using SPSS 26.0 software. Measurement data following a normal distribution were expressed as mean x *± s*, while data with a non-normal distribution were presented as median (interquartile range) [M (Q1, Q3)]. Comparisons between groups were conducted using the *Mann-Whitney U* test. Categorical data are reported as counts and percentages. All tests were two-tailed, and *P* < 0.05 was considered statistically significant.

This study was approved by the Medical Ethics Committee of the University of Hong Kong Shenzhen Hospital, under project number HKUSZH2022021.

## Results

### Patient characteristics

A total of 117 patients underwent total arterial coronary artery bypass grafting surgery, comprising 102 males and 15 females, with an average age of (60.0 ± 10.0) years. Patients received an average of (3.1 ± 0.6) bypass grafts each. The average operation time and postoperative ventilator time were (6.5 ± 1.2) hours and (7.7 ± 3.6) hours, respectively. During the perioperative period, 21 patients required IABP assistance. The clinical data are presented in Table 1.

**Table 1:** Clinical characteristics

Characteristic	Data
Gender (male/female)	102/15
Age (yrs, x *± s*)	59.6 ± 10.2
Height(cm, x ± s)	165.9 ± 6.8
BMI	23.6 ± 3.4
Smoking [n (%)]	40(34.2)
Hypertension [n (%)]	77(65.8)
Diabetes [n (%)]	51(43.6)
Hyperlipidemia [n (%)]	62(53.0)
Family history of coronary heart disease [n (%)]	3(2.6)
Preoperative serum creatinine (umol/L, x ± s)	103.7 ± 96.3
Preoperative dialysis [n (%)]	2(1.7)
Cerebrovascular disease [n (%)]	13(11.1)
Carotid artery stenosis [n (%)]	19(16.2)
Peripheral vascular disease [n (%)]	9(7.7)
Lung disease [case (%)]	3(2.6)
Acute myocardial infarction [n (%)]	25(21.4)
Unstable angina pectoris [n (%)]	57(48.7)
NYHA grade > I [n (%)]	40(34.2)
Left main stenosis [case (%)]	38(32.5)
Number of diseased coronary arteries (x ± s)	2.8 ± 0.4
EF < 50% [n (%)]	24(20.5)
Surgery information	
OPCAB [n (%)]	89(76.1)
Grafts (n, x ± s)	3.1 ± 0.6
Surgery time (min)	269.0±46.5
Early outcome after CABG	
Deep sternal infection [n (%)]	1(0.8)
Second hemostasis [n (%)]	2(1.7)
Stroke [n (%)]	3(2.6)
Perioperative myocardial infarction [n (%)]	0(0)
Sepsis [n (%)]	0(0)
Low cardiac output [n (%)]	1(0.8)
Death within 30 days [n (%)]	0(0)

### Grafts and target vessel selection

The left internal mammary artery was used in situ in 108 cases, while in 4 cases, it was redirected to the ascending aorta due to length limitations. Bilateral radial arteries were utilized in 88 patients, and bilateral internal mammary arteries in 4 patients. The target vessels for the left internal mammary artery were primarily the left anterior descending branch, while the radial artery targeted the obtuse marginal, diagonal, intermediate, posterior descending, left ventricular posterior branches, or right coronal trunk. All proximal radial arteries were anastomosed to the aorta. A distal T-graft was created in 42 cases, and a sequential graft in 4 cases. Details of the bypass grafting and target vessels are shown in Table 2.

**Fig. 1:**
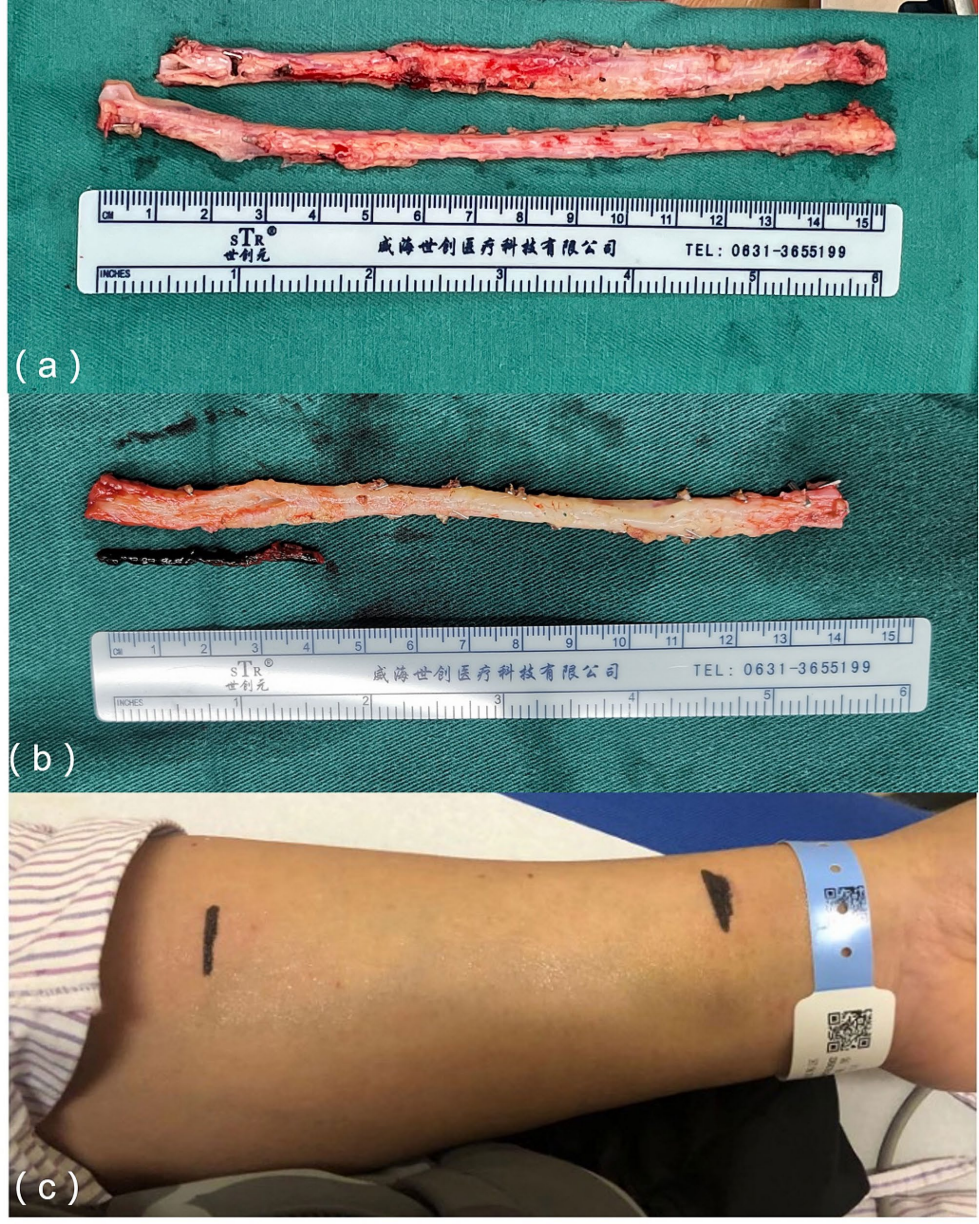
(**a**) The radial artery was harvested using the “NO-TOUCH” technique, and the target blood vessel was selected based on the measured length; (**B**) The intima of the remaining bypass grafting vessel remained intact after the distal thrombus was removed and flushed from the distal end of the radial artery; (**C**) Preoperative evaluation of the radial artery intima and lumen diameter was conducted via ultrasound and marked on the body surface

The radial artery had an average length of approximately 15 cm and was anastomosed to the appropriate target vessel based on its length (Fig. 1A). In this cohort, 2 patients had a thrombus at the distal end of the right radial artery. After removal of the thrombus, the remaining vessel intima was intact and suitable for use in the bypass graft (Fig. 1B). Before surgery, these patients underwent ultrasound examinations to assess the radial artery lumen diameter and intima smoothness, and the results were marked on the body surface. The vessels were harvested only when the required length was available. If the length of the graft vessel was insufficient during surgery, the accompanying veins of the radial artery could be excised and appropriately extended.

**Fig. 2:**
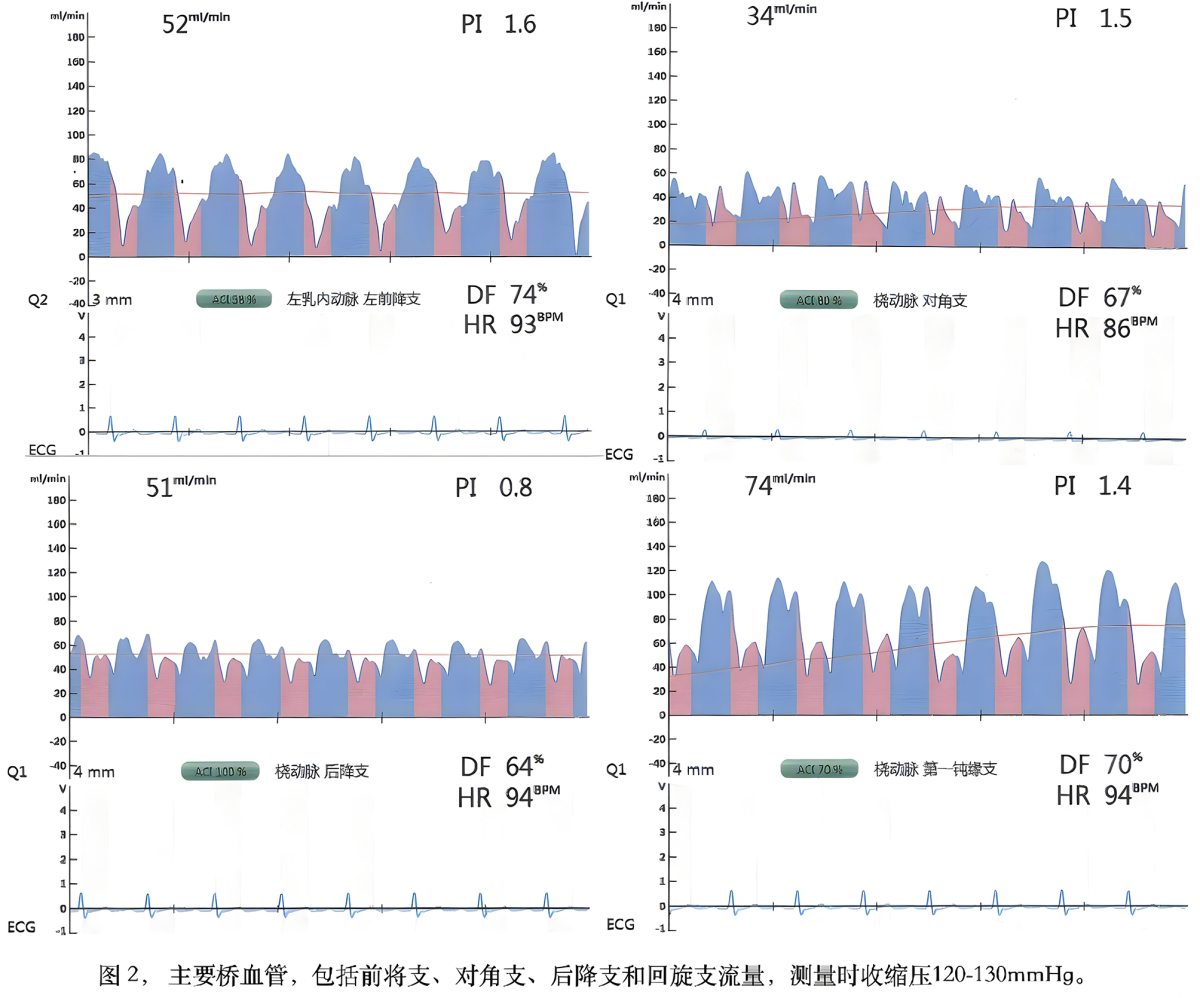
Measurement of flow in the main target vessels post-operation, with systolic blood pressure between 120-130mmHg and diastolic blood pressure 60-70mmHg during measurement

The mean flow rates of the main bypass grafting vessels measured during surgery were (36.0 ± 24.8) ml/min in the left anterior descending branch, (30.0 ± 21.0) ml/min in the obtuse marginal branch, (25.8 ± 16.3) ml/min in the posterior descending branch, and (29.3 ± 17.80 ml/min in the diagonal branch (Fig. 2).

**Table 2:** Different bypass grafting vessel used in the surgery

Bypass grafting vessel	Target vessel	Data
Left Internal mammary artery in situ	Anterior descending branch	98
	Diagonal branch	5
	Circumflex branch	1
	Obtuse Marginal Branch	4
Free internal mammary artery	Anterior descending branch	2
	Diagonal branch	2
	Obtuse Marginal Branch	4
	Right main coronary artery	1
Radial artery	Anterior descending branch	11
	Diagonal branch	41
	Obtuse Marginal Branch	89
	Right main coronary artery	19
	Posterior descending branch	68
	Posterior branch of left ventricle	11
	Intermediate branch	5
	Circumflex branch	5
	Marginal branch	1

### Pathological examination

For patients who used the radial artery as a bypass graft, the bio-bank in our hospital preserves all distal tissue samples of the radial artery, which can be used for pathological examination. Radial artery samples were selected at 1 week and 2 weeks after angiography for pathological examination. The purpose is to confirm that the radial artery intima evaluated by ultrasound is smooth and can be used as a bridging vessel.

**Fig. 3:**
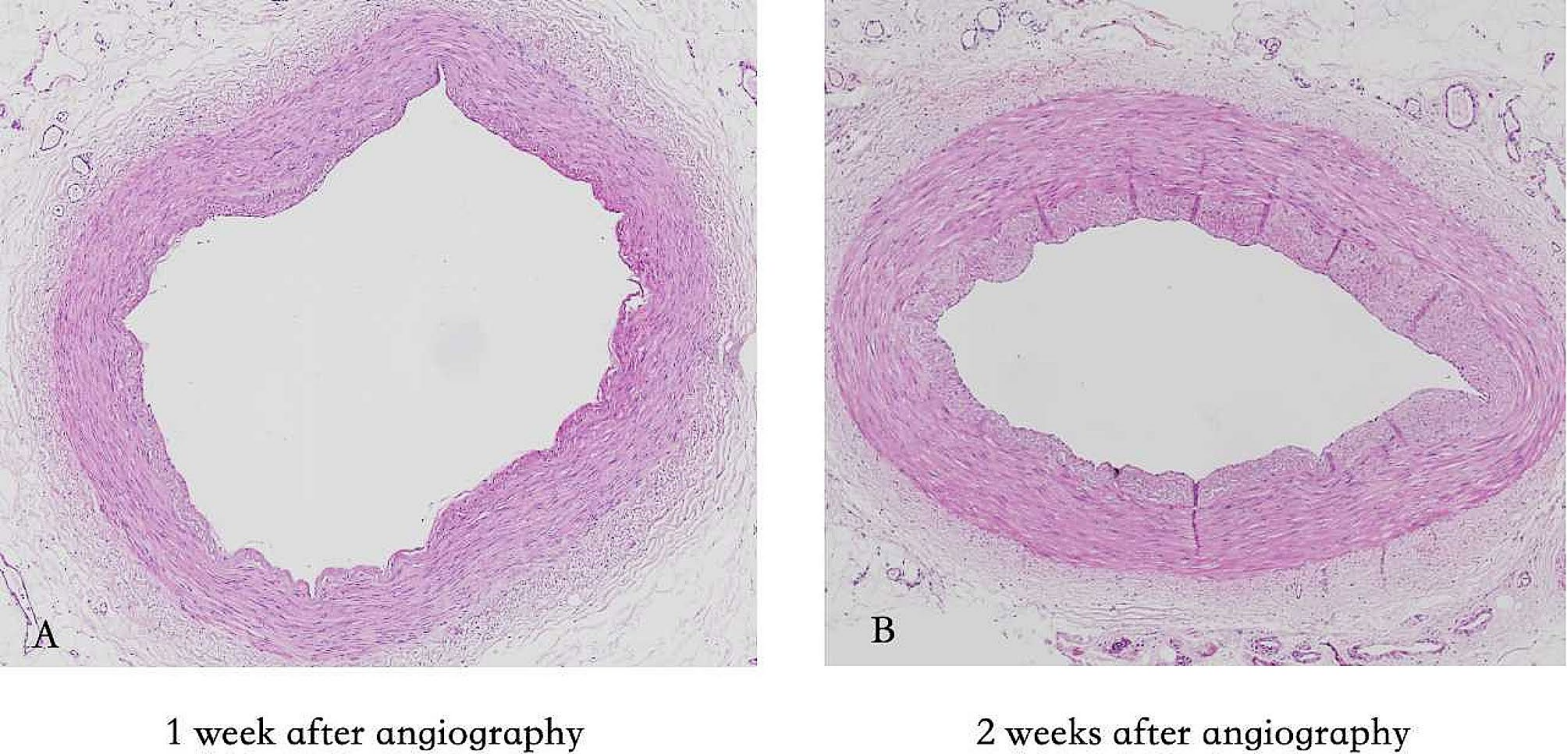
HE indicated that the radial arteries maintained intact intima, continuous elastic membranes, no significant infiltration of inflammatory cells and matrix-like components, and no lumen stenosis caused by hyperplasia one and two weeks after angiography

The HE is staining showed that the radial arteries exhibited intact intima, a continuous elastic membrane, no significant infiltration of inflammatory cells or matrix-like components, and no lumen stenosis due to hyperplasia (Fig. 3). These findings were consistent with those from ultrasound evaluations.

### Follow-up

There were no in-hospital deaths after the surgery. During the perioperative period, 3 patients experienced cerebral infarctions but were discharged after receiving rehabilitation treatment. Two patients required a second thoracotomy to address bleeding, and 1 patient experienced delayed wound healing, which improved significantly after debridement. Two weeks post-operation, coronary CTA was performed to reexamine blood flow. No occlusions were observed in the internal mammary arteries, but 4 cases exhibited radial artery occlusion. Among these, 3 target vessels were the posterior descending branches (2 with 70% stenosis and 1 with 100% stenosis), and the remaining target vessel was the obtuse marginal branch (with 60% stenosis), forming a T-shaped graft with the diagonal branch at 90% stenosis. The patients exhibited no symptoms of angina pectoris and received no specific treatment. Follow-up ranged from 2 to 25 months post-discharge, with no deaths or new cardiovascular or cerebrovascular events reported. Throughout the follow-up period, the survival rate without major cardiovascular and cerebrovascular events (cardiovascular death, myocardial infarction, and stroke) remained at 100%.

## Discussion

Recent studies have shown that the radial artery has higher patency rates than the great saphenous vein during early and middle follow-up periods [11, 12]. Many patients with coronary heart disease complicated with diabetes. It has been confirmed that the use of bilateral internal mammary arteries will increase the risk of poor sternal healing and infection. At the same time, the radial artery, as a graft, has a higher long-term patency rate than the great saphenous vein, faster wound healing in both upper limbs, and fewer complications.

However, it has been reported that endothelial damage at the puncture site of the radial artery after coronary angiography can take over 4 weeks to recover [13]. With the improvement of puncture techneque and gentle operation, the postoperative recovery of the radial artery intima is faster. Moreover, new techneques icluding the use of thinner sheaths, sheaths less likely to cause vascular damage, or innovative techniques in guidewire insertion, are helpful to accelerate its recover. Furthermore, it was reported that distal artery puncture was safe. And distal radial access prevents radial artery occlusion [14].

In this study, a certain part of patients displayed a smooth and fully recovered intima within 7–14 days post-angiography, as confirmed by pathology and ultrasound evaluations. The preoperative assessment of the radial artery was also refined. In addition to the clinical ALLEN test, the ultrasound Doppler examination was enhanced by introducing additional measurement indices to provide a more accurate evaluation of the radial artery, especially after use of the right radial artery for angiography. (1) Evaluation of radial artery diameter: measurements were taken at the radial styloid process, 2 cm below the elbow fossa, and the midsection of the radial artery to accurately assess vessel thickness and determine suitability for use as a bypass graft. (2) Ultrasonic ALLEN test: Traditional clinical ALLEN tests can be influenced by factors such as the completeness of radial artery compression, skin color, and lighting conditions, potentially leading to inaccuracies [14]. In this study, ultrasound was employed to perform the ALLEN test on all patients, eliminating these variables. A 30% increase in ulnar artery blood flow was considered indicative of adequate collateral circulation in the palm. (3) Evaluation of blood vessel intima: In addition to diameter, evaluating the intima of the artery is crucial. As age increases, plaques or stenosis may develop in limb blood vessels. Ultrasound is useful for identifying calcified plaques and severe stenosis in the radial artery, thus helping to avoid potential impacts on long-term patency rates. (4) Evaluation of the radial artery after angiography: Most patients scheduled for coronary artery bypass grafting underwent the procedure within 1–2 weeks after angiography. The intima appeared intact in Doppler ultrasound examinations of patients with successful puncture. Due to puncture and other factors, some patients showed thromboembolism or irregular intima at the distal end on ultrasound, while a few exhibited recanalization of the radial artery with an intact intima when awaiting surgery. For patients with distal embolism, ultrasound was able to determine the length of unobstructed radial artery and confirm its suitability as a graft if the length was sufficient. Pathological sections and HE staining corroborated these findings, showing intact intima, continuous elastic membranes, and the absence of significant inflammatory cell infiltration, matrix-like components, or hyperplasia-induced lumen stenosis in both left and right radial arteries. These results confirm that ultrasound is an effective method for preoperative evaluation of the radial artery intima, providing substantial evidence for its clinical use.

Spasm remains a primary concern regarding the use of the radial artery as a graft [15, 16]. Various strategies, including the “NO-TOUCH” technique, have been employed to prevent perioperative spasm. This technique involves gentle handling, avoiding excessive traction, and selecting highly narrow target blood vessels to increase patency rates [17, 18]. In this study, the application of the radial artery in bypass grafting procedures achieved satisfactory short-term results by optimizing preoperative evaluations and the grafting process. These findings provide initial evidence supporting the safety, reliability, and potential future use of radial artery bypass grafting in clinical practice. The surgical procedure was refined to optimize the bypass sequence: Firstly, the radial artery was anastomosed to the ascending aorta, ensuring unobstructed blood flow. The residual thrombus in the radial artery could be expelled by the blood flow of the ascending aorta. Subsequently, the distal radial artery was clamped to prevent bleeding and to allow the artery to adapt to the ascending aortic pressure. Previous studies have shown that the radial artery has a thick wall and abundant vascular smooth muscle cells, which may contribute to its susceptibility to spasm [19, 20]. We hypothesize that after anastomosis to the ascending aorta, the radial artery can withstand pressure for a certain period, effectively reducing or avoiding spasm.

Maintaining effective perfusion pressure is another crucial factor in avoiding vessel spasm. In our study, patients with perioperative systolic blood pressure maintained above 120mmHg and average pressure above 70mmHg experienced no perioperative vasospasm or major cardiovascular events. Furthermore, our facility regularly provided norepinephrine and epinephrine for post-operative blood pressure management, although calcium antagonists like diltiazem were not specifically utilized. These findings demonstrate that adapting to ascending aortic pressure after completing proximal radial artery anastomosis, pre-expanding the vessel, and maintaining effective perfusion pressure are important measures for preventing or reducing perioperative spasm. It is worth noting that a relatively high proportion of patients in our study required intra-aortic balloon pump (IABP) assistance. Since the off-pump technique was employed for CABG to facilitate the procedure, patients with a left ventricular ejection fraction (EF) value of ≤ 40% before the operation received IABP assistance to maintain perioperative hemodynamic stability, ensure coronary perfusion pressure, and prevent radial artery spasm.

It is important to recognize that this study has many constraints. Firstly, the sample size was relatively small, which may limit the generalizability of the findings. Secondly, there was an absence of extended follow-up data, and no postoperative coronary angiography was conducted. Although a coronary computed tomography angiography (CTA) was conducted two weeks after the operation, revealing four undeveloped radial arteries, it is important to consider that the contrast agent may not have passed through the radial artery due to potential competitive blood flow. Subsequent examination is necessary in future studies to tackle these constraints.

## Conclusion

Based on the initial findings of this study, it has been observed that radial artery can regain its structural integrity within a period of 7–14 days following coronary angiography in certain patients. An optimal ultrasonic examination technique can be used to adequately evaluate this restoration process. However, the survival rates and the patency rates of the grafts in the postoperative phase need to be verified through more extensive prospective investigations.

## Data Availability

No datasets were generated or analysed during the current study.
